# Enzyme structure dynamics of xylanase I from *Trichoderma longibrachiatum*

**DOI:** 10.1186/1471-2105-11-S6-S12

**Published:** 2010-10-07

**Authors:** Ugur Uzuner, Weibing Shi, Lantao Liu, Sanmin Liu, Susie Y Dai, Joshua S Yuan

**Affiliations:** 1 Department of Plant Pathology and Microbiology, Texas A&M University, College Station, TX 77843, USA; 2 Institute for Plant Genomics and Biotechnology, Texas A&M University, College Station, TX 77843, USA; 3 Department of Veterinary Pathobiology, Texas A&M University, College Station, TX, 77843, USA; 4 Office of the Texas State Chemist, Texas A&M University, College Station, TX 77843, USA; 5 Advanced Research Institute of Sustainable Energy, Texas A&M University, College Station, TX, 77843, USA

## Abstract

**Background:**

Enzyme dynamics has recently been shown to be crucial for structure-function relationship. Among various structure dynamics analysis platforms, HDX (hydrogen deuterium exchange) mass spectrometry stands out as an efficient and high-throughput way to analyze protein dynamics upon ligand binding. Despite the potential, limited research has employed the HDX mass spec platform to probe regional structure dynamics of enzymes. In particular, the technique has never been used for analyzing cell wall degrading enzymes. We hereby used xylanase as a model to explore the potential of HDX mass spectrometry for studying cell wall degrading enzymes.

**Results:**

HDX mass spectrometry revealed significant intrinsic dynamics for the xylanase enzyme. Different regions of the enzymes are differentially stabilized in the *apo* enzyme. The comparison of substrate-binding enzymes revealed that xylohexaose can significantly stabilize the enzyme. Several regions including those near the reaction centres were significantly stabilized during the xylohexaose binding. As compared to xylohexaose, xylan induced relatively less protection in the enzyme, which may be due to the insolubility of the substrate. The structure relevance of the enzyme dynamics was discussed with reference to the three dimensional structure of the enzyme. HDX mass spectrometry revealed strong dynamics-function relevance and such relevance can be explored for the future enzyme improvement.

**Conclusion:**

Ligand-binding can lead to the significant stabilization at both regional and global level for enzymes like xylanase. HDX mass spectrometry is a powerful high-throughput platform to identify the key regions protected during the ligand binding and to explore the molecular mechanisms of the enzyme function. The HDX mass spectrometry analysis of cell wall degrading enzymes has provided a novel platform to guide the rational design of enzymes.

## Background

Xylan is the major hemicellulose component of the plant cell wall and the second most abundant natural polysaccharide. Most of xylan is a heteropolysaccharide consisting of β-1,4-linked D-xylose monomers in connection with side branches of arabinosyl, glucuronosyl, acetyl, uronyl, and mannosyl residues [1]. Complete degradation of xylan structures requires the concerted and synergistic function of several enzymes including endo- β-1, 4-xylanases (EC 3.2.1.8) [2]. Due to the broad applications in biopulping and biobleaching in paper industry, xylanase has been one of the major research focuses for bioconversion [1]. In particular, endoxylanases have been thoroughly studied as the major lignocellulosic biomass degradation enzymes. Xylanases with high substrate binding specificity, enhanced enzymatic activity, and increased thermostability are needed for various biorefinery applications. Tremendous efforts have been devoted to improve xylanase and cellulase enzyme performance by manipulating the protein amino sequence in the past [3]. However, sequence-based protein modification has its limitations [4,5]. It is experimentally infeasible to test all possible mutants of a protein, and it is time consuming since the majority of the manipulated sequences do not fold properly into functional proteins [6]. Suitable techniques thus are needed to guide enzyme improvement with structure-function relationship for better enzyme rational design and engineering.

Structure dynamics has become an important consideration for enzyme engineering [7-14]. The structure dynamics during the enzyme catalysis can be derived from molecular dynamics simulations, NMR, and mass spectrometry-based methods. In particular, novel HDX mass spectrometry platforms provide the structure dynamics information for enzyme engineering [15,16]. Recent research unveiled how structure dynamics is related to enzyme function [9,13,16]. The structure dynamics-guided approach has been successfully used for enzyme activity improvement [7,8]. Hydrogen/deuterium exchange mass spectrometry (HDX-MS) represents one of the most widely used platforms for exploring protein conformational dynamics, folding, and binding [17-22]. HDX mass spectrometry has been broadly applied to study protein dynamics and structure, in particularly for the protein binding with ligands, substrates, DNA and other molecules [23-27]. Such analysis has enabled the illustration of the enzyme substrate interaction mechanism and the protein binding molecular determinants [28,29].

The fundamental concept of HDX mass spectrometry analysis is based on the mass increase of a protein when the protein protons exchange with the solvent deuterium [30]. The rate and percentage of the H/D exchange can be measured by mass to charge ratio (m/z) of the protein. The HDX mass spectrometry can be used to study the global and regional protein conformational changes with different platforms [31,32]. Coupled with protein digestion and chromatography separation, the HDX mass spectrometry is able to profile different regions of protein for H/D exchange based on the peptide H/D exchange rate and percentage. The underlying cause of HDX structure dynamics may involve changes in hydrogen bonds and other forces [27]. For instance, if the protein binding with ligand leads to more H/D exchange in a region, the ligand binding is expected to induce conformational changes to destabilize the region. HDX mass spectrometry thus allows us to probe the protein structure dynamics changes during enzymatic reactions [9,28,33].

The HDX platform comes with the advantages of mass spectrometry analysis: fast, straightforward, and environmentally friendly [27]. The HDX mass spectrometry technologies thus provide user-friendly alternatives to study the structure and dynamics of xylanase in a way that is not possible with other technologies. The advantages of HDX mass spectrometry over X-Ray and NMR are higher throughput, less protein purity requirements, and the dynamics and stability information rendered [34,35]. Compared with X-Ray or NMR techniques, HDX mass spectrometry is difficult to resolve structures at a single amino acid residue resolution. The resolution of the techniques relies on the protease digestion, which will produce peptides of varied length in a protein-dependent manner. In addition, the conformational changes observed are the backbone changes, and the side chain information is limited. However, it is of great interest to resolve peptide regions that span several amino acid residues to localize the stabilized or destabilized region during the catalysis or inhibition. The backbone changes contribute to the conformational changes involved in enzyme reactions [24,28]. The combination of HDX mass spectrometry with X-ray data and computational modelling is a potent way to provide more detailed information regarding the structure, stability, and dynamics of enzyme/substrate or enzyme/inhibitor interactions.

In the present study, the structure dynamics changes of the* Trichoderma longibrachiatum* xylanase enzyme upon binding with xylohexaose and xylan ligands was evaluated with HDX mass spectrometry analysis. The analyses revealed important regional dynamics of xylanase upon ligand binding. Combination of the structure dynamics data from the HDX analyses and the static structure information from the X-ray crystallography provided novel insights for further enzyme improvement.

## Results and discussion

### Data processing for HDX mass spectrometry

**Figure 1:**
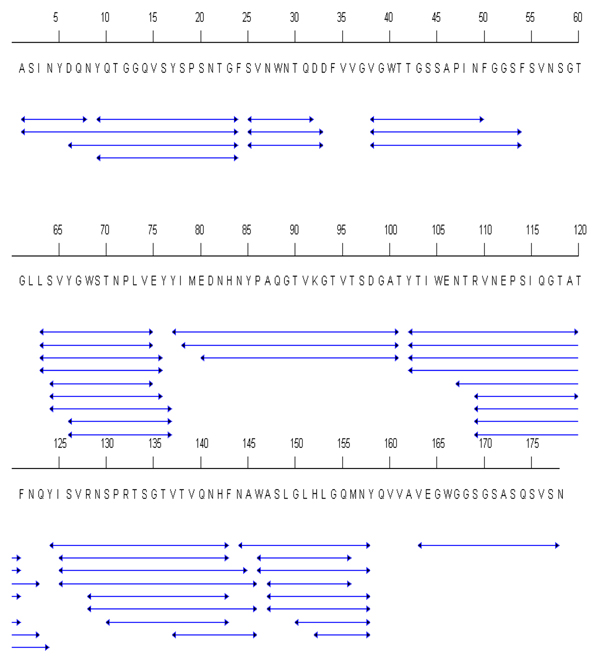
**Peptides analyzed in the HDX experiment for xylanase (1XYN chain A).** The blue arrowed lines donate the sequence of the peptide.

**Figure 2:**
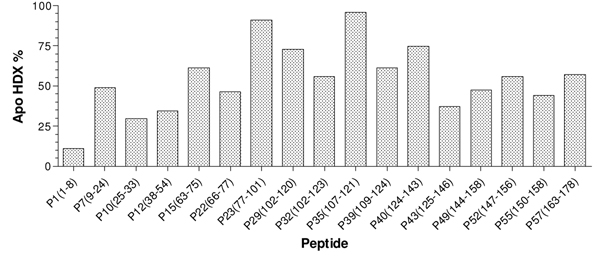
**HDX percentage of* apo* xylanase for representative peptides.** The selected peptides have a total of 91% sequence coverage. The percentage is the averaged value of five HDX experiments with different exchange times (60, 240, 960, 1920, and 3840 seconds).

**Figure 3:**
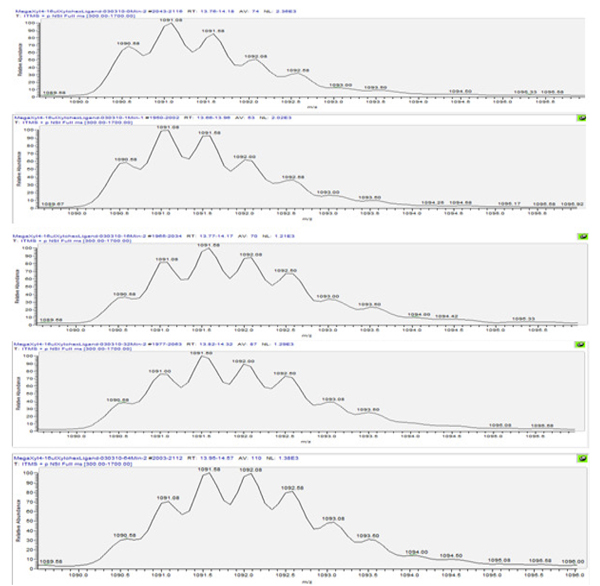
**The mass spectra of peptide ‘YTIWENTRVNEPSIQGTAT’ containing residues from 102 to120.** The HDX exchange time from top to bottom is 60, 240, 960, 1920, and 3840 seconds, respectively. The shift of the peak centroid toward to the left indicated incorporation of deuterium into the backbone of the region.

The xylanase enzyme of* T. longibrachiatum* shared 100% sequence identity to that of* T. reesei.* A total of 57 digested peptides were identified in the MSMS data acquisition (Additional file 1) with the sequence coverage of 91% (Figure 1). In addition, the HDX analyses rendered 45 peptides with significant signal to noise ratio (S/N>3) and gave 71% sequence coverage. The covered region is given in Figure 2. Twelve peptides that were identified in the MSMS acquisition could not be measured accurately in the HDX experiments due to co-elution problems or weak signal after long exchange times (for precise description, see Additional file 1). The representative deuterium incorporation spectra were illustrated in Figure 3 for peptide ‘YTIWENTRVNEPSIQGTAT’ (residues; 102-120).

### Structure dynamics revealed by HDX analysis in* Apo* protein

The HDX analysis of* apo* xylanase showed that the enzyme dynamics is regional specific (Figure 2). Regions that include residues 77 to 101 and 107 to 121 showed the greatest hydrogen deuterium exchange in the* apo* enzyme, and the N-terminal of the enzyme is less dynamic according to the overall hydrogen deuterium exchange. Overall, the enzyme is dynamic based on the HDX experiment considering that the average exchange percentage is 50 % for all five exchange times (i.e. 60, 240, 960, 1920, and 3840 seconds).

### Differential HDX analysis of xylanase

**Figure 4:**
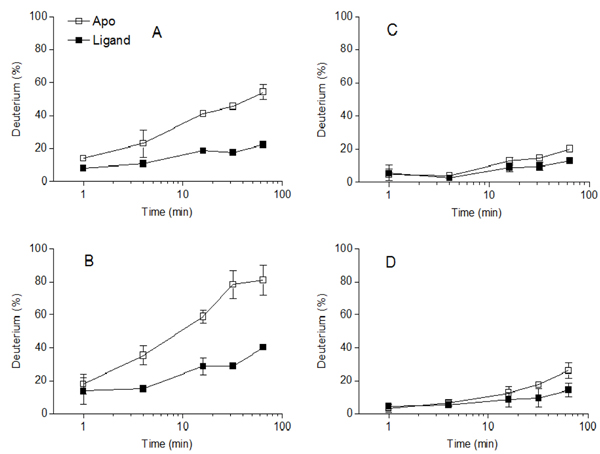
**Deuterium incorporation curves of four peptides.** HDX data for the* apo* xylanase is represented by the open square, and HDX data for the xylanase interacting with xylohexaose substrate is represented with the closed square. A. HDX data for peptide ‘VGWTTGSSAPINF’ containing residues from 38 to 50. B. HDX data for peptide ‘YTIWENTRVNEPSIQGTAT’ containing residues from 102 to 120. C. HDX data for peptide ‘SVYGWSTNPLVEY’ containing residues from 64 to 76, D. HDX data for peptide ‘SVYGWSTNPLVEYY’ containing residues from 64 to 77.

Differential HDX analysis of the* apo* protein versus the protein ligand complex revealed the structure dynamics change of the enzyme upon ligand binding. Figure 4 gave two examples that regions were protected in the HDX experiment. The protected region had less deuterium incorporation with the same exchange time. Peptide ‘VGWTTGSSAPINF’ in Figure 4A (residues; 38-50) and peptide ‘YTIWENTRVNEPSIQGTAT’ in Figure 4B (residues; 102-120) showed significant protection in the HDX measurements. Contrary to that, peptide ‘SVYGWSTNPLVEY’ (residues; 64-76), and peptide ‘SVYGWSTNPLVEYY’ (residues; 64-77) showed similar exchange pattern in the* apo* protein and holo protein, which suggested those regions had little structure dynamics change upon xylohexaose binding. More importantly, the two peptides had only one amino acid differences and had an essentially similar HDX profile, which highlighted the reproducibility of our data. The differences in the structure dynamics probed by hydrogen deuterium exchange potentially reflected the differential local structure protections. The common speculation of mechanism for hydrogen/deuterium exchange involves hydrogen bonding and possible solvent accessibility, which lead to different hydrogen/deuterium exchange rates in different protein regions [36]. Nevertheless, the HDX analysis revealed the differential structure dynamic of different xylanase regions when binding with substrate xylohexaose.

### Statistical analysis for differential HDX data

**Figure 5:**
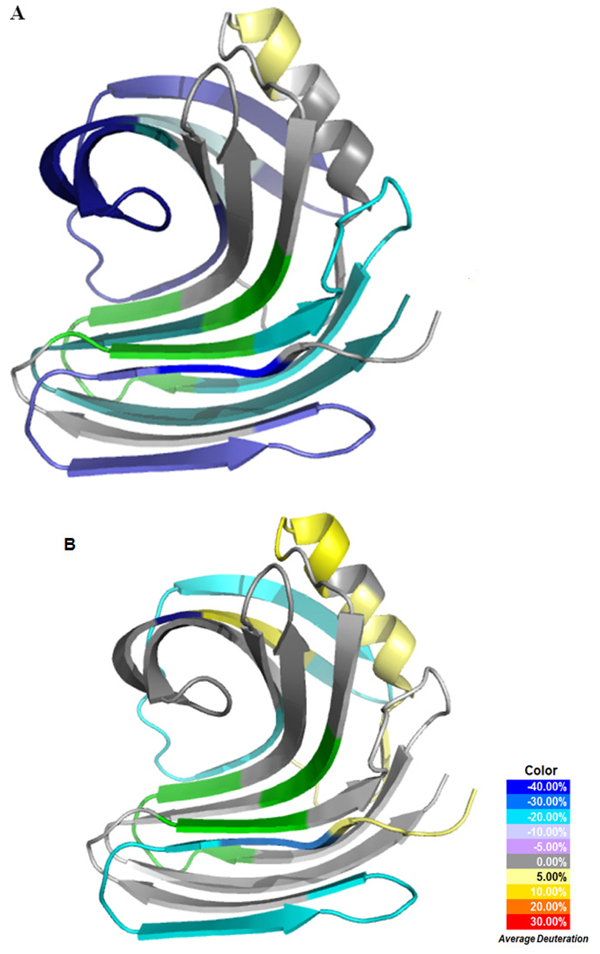
**HDX profile overlaid onto xylanase crystal structures.** A) HDX profile of xylanase/xylohexaose complex overlaid onto the* apo* xylanase crystal structure (PDB: 1XYNA), B) HDX profile of xylanase/xylan complex overlaid onto* apo* xylanase crystal structure (PDB:1XYNA), The color legend shows the deuterium incorporation difference by subtracting deuterium incorporation content of* holo* xylanase from* apo* xylanase. The green coloured regions represent peptides that are not detected after pepsin digestion or cannot be measured accurately in the HDX experiments due to co-elution problems.

Despite the visualization in Figure 4, the differential HDX profile needs to be determined statistically. We chose the last three time points for the paired student* t* test analysis to compare the* apo* and ligand binding mass spectra centroid value as shown in Additional file 1. For the* apo* enzyme, the maximum D_2_O exchange percentage (HDX percentage) for each peptide ranged from 10% to 95.7%. In parallel, HDX percentage was also defined for protein binding with the substrate. The level of protection for a particular peptide can be determined by two methods. First, we can calculate the differences for the HDX percentage between the* apo* and* holo* protein. Second, we can calculate the differences of the centroid of the mass spectra for the peptides. The advantage for the percentage calculation is that it gives a numeric value that is normalized against the size and the charge state of the protein. The advantage of centroid value is that it is the direct measurement of the HDX mass spectra and thus can be more readily subject to various statistical analyses. We therefore combined the strength of the two types of measurement. For the visualization in Figure 5, we adopted the percentage differences. For the statistical analysis in Additional file 1, we included the paired student’s t-test of mass spectra centroid for peptides to compare the* apo* and* holo* proteins.

The results revealed that the substrate xylohexaose played an important role in the stabilization of the xylanase chain A, because the HDX rate for most of the peptides were smaller than* apo,* and the centroid values were significantly lower than those of* apo* protein for most peptides (P <0.01). Only a few peptides such as the peptide ‘SVNWNTQDD’ and ‘NTRVNEPSIQGTATF’ showed no significant differences in peptide centroid between the* apo* and* holo* protein (*t* =0.9553 and 0.9681,* P* =0.3627 and 0.3614).

In contrast, most of the peptides were not significantly stabilized by xylan binding. Upon the xylan binding, only a few peptides including ‘SVNWNTQDD’ (*t*=4.76, *P*<0.01), ‘LSVYGWSTNPLVEY’ (*t*=3.08, *P*=0.02), ‘YGWSTNPLVEYY’ (*t* =4.52,* P* <0.01), ‘ISVRNSPRTSGTVTVQNHF’ (*t* =3.23,* P* =0.01), and ‘NAWASLGLHLGQMNY’ (*t*=2.81,* P* =0.02) was significantly stabilized by xylan as shown by the statistical test in Additional file 1. Even though some peptide exhibited large HDX percentage differences, these peptides did not show significant differences in the centroid values. The results highlighted the importance of statistical analysis in the interpretation of structure dynamics.

### Correlation of HDX data with X-ray structure

Correlation of the HDX data with the X-ray three dimensional structure data can also provide useful information to understand the enzyme function. The 3D structure of xylanase from* T. reesei* was used as the template to overlay the structure dynamics with the static structure, because the enzyme shares 100% amino acid sequence identity with the* T. longibrachiatum* Xylanase. Excellent consistency between the X-ray structure and the HDX analyses was observed. The active sites of XYN1 Glu75, Glu164 are located in B6 and B4 β strands [37]. The protein was described to have the analogy of the right hand. As shown in Figure 5A, the HDX profile revealed that the regions close to the reaction center had the highest protection when the enzyme bound with xylohexaose ligand. Considering that enzyme reaction often involves the substrate interaction with the reaction sites, leading to the stabilization in the region, the results highlighted the potential regions essential for the ligand binding process and substrate specificity (Figure 5A). In addition, the thumb region of the protein as suggested in the previous publication [37] was highly stabilized during the ligand binding. The stabilization indicated that the thumb region of xylanase might also play an important role in binding and processing of the xylohexaose substrates. HDX analysis thus had the potential to reveal important regions for substrate binding and enzyme reaction beyond the reaction sites. Besides the thumb regions, we also identified some other stabilized regions outside of the substrate binding groove. For instance, the peptide containing residues from 77 to101 located only one residue away from one of the active sites and exhibited the greatest protection in the HDX experiment. The protection was also observed with the peptide containing residues from 6 to 24, which consists one of the fingers of the right hand structure of the protein.

### Comparative analysis of structure dynamics when binding with two substrates

As aforementioned, we analyzed the HDX profile for two substrates in our study, xylan and xylohexaose. The results showed rather different structure dynamics induced by different substrate binding as shown Figure 5 and Additional file 1. Overall, there seemed to be less enzyme structure dynamics changes for xylan binding than those for xylohexaose binding. Even though the differences in HDX percentage as shown in Figure 5B still showed some regions of changes, most of peptide did not show a statistically significant change in the HDX percentage. The results along with the HDX profile indicated a high variation for the HDX percentage measurement. Such variation between the substrates is due to the solubility of the xylan. Xylan is not entirely soluble and the solution forms an emulsion during the experiment, which might have led to the significant variation of the HDX analysis during the xylan binding experiments. The results indicated the xylohexaose binding might have rendered much more reliable data for mechanism studies.

### HDX profile and enzyme improvement for the future

Engineering protein flexibility (plasticity) can be used to enhance substrate/ligand specificity of the protein by increasing the rigidity of flexible residues [38]. For this reason, we carried out the HDX analysis for xylanase to identify the critical sequences that could be stabilized to strengthen the local interactions of the groove region and neighboring residues. Our data provided important information regarding the global and local dynamics of xylanase enzymes when binding with different substrates. We aim to use the structure dynamics information to guide future enzyme engineering. The highly stabilized regions are believed to be important for enzyme-substrate interaction and thus are the regions of choice for downstream protein engineering work.

**Figure 6:**
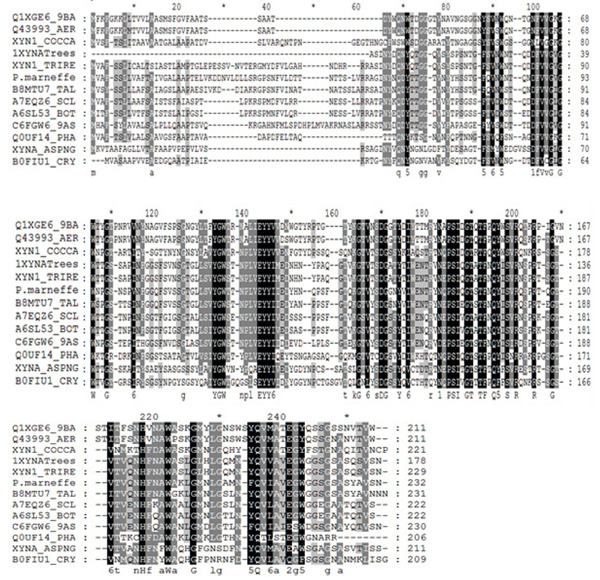
**Multiple sequence alignment of homologs of *T.****longibrachiatum* xylanase. The sequence alignment includes only XYN1 sequences from different organisms. The sequences are endo-1,4-beta-xylanase for* Paenibacillus sp.* (Q1XGE6_9BA), *Aeromonas punctata* (Q43993_AER),* Cochliobolus carbonum* (XYN1_COCCA),* T. reesei* (XYN1_TRIRE),* P. marneffe* (P. Marneffei),* Talaromyces stipitatus* (B8MTU7_TAL),* Sclerotinia sclerotiorum* (A7EQZ6_SCL), and* Botryotinia fuckeliana* (A6SL53_BOT),* Bispora sp.* (C6FGW6_9AS),* Phaeosphaeria nodorum* (Q0UF14_PHA), A.* niger* (XYNA_ASPNG), and* Cryptococcus flavus* (B0FIU1_CRY).

In order to relay the HDX analysis result to enzyme evolution and improvement, we carried out the multiple sequence alignment of xylanase enzymes from different species and families as shown in Figure 6. Ten fungal and two bacterial xylanase 1 sequences were aligned together with the* T. longibrachiatum* xylanase. The multiple sequence alignment revealed that some highly conserved regions like peptide ‘RVNEPSIQGTATFNQY’ (residues; 109-124) corresponded to the regions significantly stabilized during the substrate binding. This peptide consists of the thumb region of the xylanase and locates very close to the active site (Glu75) of the protein (Figure 5A and 5B; Figure 6). The significant protection indicated its crucial role in substrate recruitment or holding. The evolutionary evidence and structure dynamics information correlated with one another indicating the region to be one of the structure dynamics determinants for enzyme function. Besides the peptide containing residues from 109 to 124, many other regions were shown to be significantly protected during the substrate binding, and some of these regions were less conserved. We are designing domain swapping experiments to modify various regions for enzyme improvement and mechanistic study.

In industrial applications, the hydrolysis step of the lignocellulosic biomass processing requires unique functions of enzymes that can be highly efficient with extreme pH tolerance, increased thermostability, and improved substrate specificity. HDX mass spectrometry provided a powerful platform to study enzyme dynamics at different conditions, and thus could be used to guide the enzyme improvement for various features. We are using the HDX analysis to explore a broader range of cell wall degrading enzymes for both mechanistic study and enzyme engineering.

## Conclusions

Overall, HDX analysis has revealed significant intrinsic dynamics for xylanase enzyme and such dynamics might be important for the enzyme function. Specifically, different regions of the* apo* xylanase showed a differential HDX rate. The substrate binding leads to significant protection or stabilization effects, where some regions near the reaction sites were significantly stabilized by the enzyme substrate xylohexaose. The xylohexaose binding also induced protection on regions beyond the reaction center. In contrary to xylohexaose, xylan binding induced fewer changes in enzyme structure dynamics, assumingly due to the insufficient binding resulted from the insolubility of xylan. The structure dynamics information for substrate binding indicated that many regions in the protein coordinatively changed conformation to fulfil the function of substrate docking and catalysis. The structure dynamics information also correlated with the enzyme evolution to a certain degree, where the evolutionarily conserved regions were very well protected by the substrate binding. These regions were expected to be essential for the enzyme function. Overall, HDX mass spec analysis allows us to identify the novel structure determinants for the enzyme function that could not be found with traditional X-ray or NMR techniques. HDX mass spectrometry thus provided a novel platform to guide the rational design of enzymes.

## Material and methods

### Protein and reagents

The endo-1,4-β-Xylanase M2 (EC 3.2.1.8) of* T. longibrachiatum* was purchased from Megazyme (Megazyme International Ireland Ltd., Wicklow, Ireland) and was used throughout the all HDX experimental processes without further purification. The protein solution was provided as a mixture of ammonium sulphate 45%, sodium azide 0.02%, water 45% and Xylanase M2 (*T. longibrachiatum*) 2.5%. The substrate, xylohexaose (molecular weight, MW: 810.70 g^−1^, Cat No: O-XHE) with >95% purity, was also provided from Megazyme. The second substrate of xylanase enzyme for this study was 2% oats spelts xylan obtained from TCI AMERICA (Portland, OR, Cat No: X0011).

### HDX experiment

HDX experiments were similar to those previously described except without using the LEAP Technologies Twin HTS PAL liquid handling robot [39,40]. Briefly, the xylanase protein solution was used without further purification at the concentration of 13.6 mg/mL in solution. The xylohexaose was dissolved in a D_2_O buffer (20mM Tris-HCL, 100mM KCL, and 1mM DTT in D_2_O, pD 7.9) to reach a final concentration of 25mM. Xylan was dissolved in D_2_O buffer to make up a 1% solution. Four µL of the xylanase solution was mixed with 16 µL of the ligand D_2_O buffer for HDX experiments at room temperature for 0, 60, 240, 960, 1920, and 3840 seconds, respectively. After the incubation in D_2_O at an aforementioned hydrogen deuterium exchange time, the exchange reaction was quenched with 30 µL ice-cold solution containing 2M urea and 1% Trifluoroacetic acid (TFA), injected into an injection valve with 50 µL sample loop, and then passed through a pepsin column (Applied biosystems, Foster City, CA) by a solvent pump (0.1% TFA in water) with flow rate at 200 µL/min. The pepsin column was kept on ice. The digested xylanase peptides were then eluted through a micro peptide cartridge (Michrom Bioresources, Inc., Auburn, CA) and desalted. The digestion and desalting takes a total of 5 min. Peptides were then eluted across a 2.1mm x 5cm C18 column (Thermo Scientific, Waltham, MA) with a linear gradient of 2%-50% Solution B over Solution A for 10 min (Solvent A, 0.1% formic acid in water; solvent B, 0.1 formic acid 80% acetonitrile, 20% water; flow rate 200 µL/min). Mass spectrometric analyses were carried out with the capillary temperature at 280 ºC using LC-LTQ mass spectrometer (Thermo Scientific, Waltham, MA). The* apo* xylanase HDX experiment was performed with the same protocol except that the D_2_O solution contained no ligand.

### Peptide identification and HDX data processing

Product ion spectra were acquired in a data-dependent MS/MS mode. The precursor ion survey scan was performed and the five most abundant ions were selected for product ion analysis. MS/MS *.raw data was first converted into *.MS2 file and then searched against the database containing xylanase using SEQUEST algorithm (Bioworks, Thermo Finnigan, CA). All peptide ion assignments were inspected manually.

The weighted average m/z values of each peptide ion isotopic cluster were calculated with the in-house developed software named as HDXanalyzer (manuscript in preparation). The deuteration level was calculated based on the following equation, and corrections for back-exchange were made based on 70% deuterium recovery and accounting for 80% deuterium content in the ion-exchange buffer.

where m/z (P), m/z (N), and m/z (F) are the centroid value of partially deuterated peptide, nondeuterated peptide, and fully deuterated peptide, respectively [41].

## Authors' contributions

Joshua Yuan and Susie Dai designed the experiments. Ugur Uzuner performed the experiments. Ugur Uzuner, Weibing Shi, Lantao Liu, and Sanmin Liu performed the data analysis. Ugur Uzuner, Susie Dai, and Joshua Yuan wrote the manuscript.

## Competing interest

The authors have no competing interest for this article.

## Supplementary Material

Additional file 1 Click here for file
